# Peritoneal dialysis: increasing global utilization as an option for renal replacement therapy

**DOI:** 10.7189/jogh.09.020316

**Published:** 2019-12

**Authors:** Anna M Zimmerman

**Affiliations:** Uniformed Services University of the Health Sciences, Bethesda, Maryland, USA

Per a newly released global report called *The Global Kidney Health Atlas*, nearly one-in-ten people worldwide have chronic kidney disease [1]. As chronic kidney disease progresses into end-state kidney disease, patients need a kidney transplant or some form of dialysis to survive. The number of patients requiring these treatments is estimated to be about 1.4 million worldwide and is growing at 8% annually [2]. Continuous Ambulatory Peritoneal Dialysis (CAPD) is the main type of peritoneal dialysis (PD) used in developing countries by >80%, while in developed countries it is slightly more than half of the population [3].

Benefits of using PD as a bridge therapy until a transplant can be performed include: can be done in home, no need to drive to a hemodialysis center, more cost-effective, reduced dietary restrictions, increased freedom perception and patient satisfaction, less hemodynamically instability during hemodialysis and possibly improved quality of life. It was found to be preferred among long-term dialysis patients [2]. Peritoneal dialysis as a method of renal replacement therapy in developing countries may be advantageous in its simplicity of therapy, reduced need for trained medical staff, and minimal requirement for technical support and electricity [2,3]. Further benefits of PD are associated with preservation of residual renal function, lower hospitalization and access intervention rates, and perhaps better short-term outcome after transplantation [4]. Survival rates at 6, 12, 24, 36, 48, and 60 months were not statistically significant among patients who have had PD and hemodialysis as noted in the United States Renal Data System 2012 Annual Report [5]. As clinical outcomes of both PD and hemodialysis have been similar, PD should be the method of choice for renal replacement therapy in patients with end stage renal disease.

Unfortunately, the global trends in rates of PD have been decreasing. While the prevalence and total number of people using PD have increased, presumptively due to the increased number of patients with chronic kidney disease needing dialysis, the trend in PD as a proportion of total dialysis has decreased from 1997 to 2008 in both developed and developing countries [3]. Some suggestions for the decline in utilization include: proliferation of hemodialysis units, private dialysis provider penetration, reimbursement rates, insufficient patient education, physician bias, resource availability, lack of local manufacturing plants, and import tax on materials. Overall, results in trends in each country were varied. There are a select few countries who are promoting peritoneal dialysis and their rates of peritoneal dialysis are increasing. This could be a promising start to a new trend of utilization of peritoneal dialysis.

A major factor to be aware of is that in general among most countries, PD is more cost-effective than hemodialysis. Per the United States Renal Data System 2012 Annual Report, the annual per patient cost of hemodialysis is around US $87 500 per year, while that of PD is around US $66 750 [5]. Increasing the use of PD in the United States from eight to 15 percent would potentially produce a savings of more than 1.1 billion to the health care system over five years [6]. The cost-effectiveness of utilizing PD to hemodialysis varied globally and also throughout the last 20 years. After taking into account all possible economic implications of therapy and controlling for patient characteristics it was found that hemodialysis was between 1.25 and 2.35 times the cost of PD in 22 countries (17 developed and 5 developing), between 0.9 and 1.25 times the cost in 15 countries (2 developed and 13 developing), and between 0.22 and 0.9 times the cost in 9 countries (1 developed and 8 developing) [5]. As the research did not include loss of patient and family member productivity and cost of transportation, these results underestimate the cost savings of PD.

**Figure:**
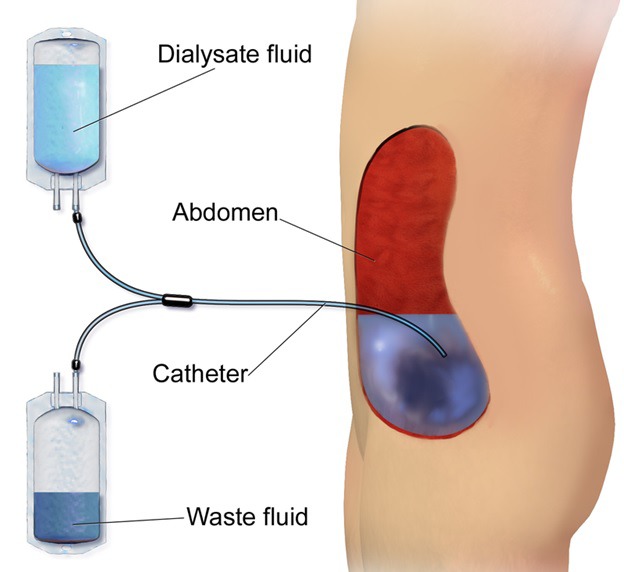
Photo: Continuous ambulatory peritoneal dialysis (CAPD). This image was donated by Blausen Medical (via WikiMedia Commons).Blausen.com staff (2014). "Medical gallery of Blausen Medical 2014". WikiJournal of Medicine 1 (2). DOI:10.15347/wjm/2014.010. ISSN 2002-4436.

The variety of cost comparisons more than likely influence utilization rates. Countries’ reimbursement structures affect utilization. In the United States, a new bundling payment system has led to an increase in PD utilization [7]. Countries that have local manufacturing plants have more cost savings using PD as materials are supplied locally and also have higher utilization rates. Countries without local manufacturers also have import taxes to pay for which negatively affects utilization. I suggest that instead of building hemodialysis centers countries should focus their dialysis program implementations on building local manufacturing centers.

A number of Asian countries have strong PD utilization rates, as they have saved 10%-30% by choosing this method [8]. Hong Kong leads the way in PD utilization rates as greater than 70% of patients use this [8,9]. Hong Kong has practiced PD ‘First Policy’ since 1985, and over 30 years has built up a model of how PD can be utilized [8]. While there should be access to hemodialysis if needed, countries around the world should strive to follow Hong Kong’s ratio as it has been shown to be sustainable.

Peritoneal dialysis should be considered the preferred method when developing renal replacement therapy programs in developing countries, as it has several benefits and is an excellent option for renal replacement therapy in patients suffering from end stage renal disease. Unfortunately, there are current clinical and economical practices globally that prevent this from being optimally utilized. Since sustainable programs have been produced and are being implemented in countries primarily in Asia, it is proven that PD can be successfully implemented in developed countries. As chronic kidney disease and end stage renal disease are going to be more prevalent globally – increasing utilization of PD is an important option to consider.

## References

[1] New global report highlights silent epidemic of kidney disease and neglect of treatment and prevention in all countries. Available: http://www.exchangemagazine.com/2017/week17/Tuesday/ 17042528.htm. Accessed: 15 May 2017.

[2] Alteri M Jindal TR Patel M Oliver DK Falta EM Elster EA PD in developing health care systems. Perit Dial Int. 2013;33:116-23. 23478372 10.3747/pdi.2012.00001 PMC3598101

[3] Jain AK Blake P Cordy P Garg AX Global trends in rates of peritoneal dialysis. J Am Soc Nephrol. 2012;23:533-44. 10.1681/ASN.2011060607 22302194 PMC3294313

[4] Pajek J Overcoming the underutilisation of peritoneal dialysis. BioMed Res Int. 2015;2015:431092. 10.1155/2015/431092 26640787 PMC4658397

[5] Karopadi AN Mason G Rettore E Ronco C Cost of peritoneal dialysis and haemodialysis across the world. Nephrol Dial Transplant. 2013;28:2553-69. 10.1093/ndt/gft214 23737482

[6] Neil N Guest S Wong L Inglese G Bhattacharyya S Gehr T The financial implications for medicare of greater use of peritoneal dialysis. Clin Ther. 2009;31:880-8. 10.1016/j.clinthera.2009.04.004 19446160

[7] How ‘Bundling’ Changed Dialysis Care. Available: http://www.renalandurologynews.com/practice-management/dialysis-bundled-payments-trends-decreased-esa-increased-peritoneal-dialysis/article/641695/. Accessed: 17 May 2017.

[8] Li P. Peritoneal dialysis: perspectives from Hong Kong, Asia Pacific and beyond. Available: https://www.thelancet.com/campaigns/kidney/updates/peritoneal-dialysis-perspectives-from-hong-kong. Accessed: 15 May 2017.

[9] Jindal RM Patel TG Waller SG Public-private partnership model to provide humanitarian services in developing countries. J Am Coll Surg. 2017;224:988-93. 10.1016/j.jamcollsurg.2016.12.056 28167227

